# Use of the Behavioral Dyscontrol Scale-2 (BDS-2) to measure executive dysfunction in FXTAS

**DOI:** 10.3389/fnmol.2026.1826779

**Published:** 2026-07-24

**Authors:** Seyedeh Ala Mokhtabad Amrei, Hasan Hasan, Ellery Santos, Hazel Maridith Barlahan Biag, Andrea Schneider, Jun Yi Wang, David R. Hessl, Flora Tassone, Ezra Morrison, Kyoungmi Kim, Randi J. Hagerman

**Affiliations:** 1 UC Davis MIND Institute, UC Davis Health, Sacramento, CA, United States; 2 University of California, Berkeley Department of Natural Resources, Berkeley, CA, United States; 3 Department of Clinical Neurosciences, Salmaniya Medical Complex, Manama, Bahrain; 4 Department of Pediatrics, University of California Davis Health, Sacramento, CA, United States; 5 Department of Psychiatry and Behavioral Sciences, University of California Davis School of Medicine, Davis, CA, United States; 6 Department of Biochemistry and Molecular Medicine, School of Medicine, University of California, Davis, Davis, CA, United States; 7 Department of Public Health Sciences, University of California, Davis, Davis, CA, United States; 8 School of Medicine, University of California, Davis, Davis, CA, United States

**Keywords:** Behavioral Dyscontrol Scale-2 (BDS-2), biomarkers, executive functioning, *FMR1* premutation, FXTAS (Fragile X-associated Tremor/Ataxia Syndrome), magnetic resonance imaging (MRI), mRNA toxicity, neurodegeneration

## Abstract

**Background/objective:**

Fragile X-associated Tremor/Ataxia Syndrome (FXTAS) is a neurodegenerative disorder caused by a premutation (55 to 200 CGG repeats) in the *FMR1* gene. This expansion leads to mRNA toxicity, resulting in the deterioration of executive and cognitive abilities alongside characteristic brain changes detected via Magnetic Resonance Imaging (MRI). While the Behavioral Dyscontrol Scale-2 (BDS-2) is commonly used to evaluate executive function, its specific relationship with neuroanatomical markers in FXTAS has not been fully examined. This study investigated the association between BDS-2 and MRI scores in FXTAS.

**Methods:**

Correlation and linear regression analyses were conducted to evaluate how BDS-2 scores correlate with MRI total scores and other neurodegeneration variables, measured by white matter hyperintensities, relative to the MMSE and CANTAB subtests in 56 *FMR1* premutation carriers with and without FXTAS.

**Results:**

MRI scores were negatively correlated with BDS-2 total scores (Pearson’s *r* = −0.66, *p* < 0.0001). The association between BDS-2 total scores and MRI scores was still significant after accounting for CGG repeat length, sex, and age as covariates (*β* = −0.36, *p* = 0.002). In the complete-case sample analysis (*n* = 30), the executive functioning subtests of the CANTAB were positively correlated with the MRI total score (*ρ* = 0.46–0.61, *p* < 0.05), while the MMSE demonstrated a negative correlation (*ρ* = −0.40, *p* = 0.03).

**Conclusion:**

These findings indicate that BDS-2 can be used alongside other instruments for measuring executive dysfunction in FXTAS, demonstrating the potential for clinical utility to complement traditional cognitive and automated executive measures.

## 1 Introduction

Fragile X syndrome (FXS) is caused by a full expansion of CGG repeats (≥200 repeats) in the 5′ region of the fragile X messenger ribonucleoprotein (*FMR1*) gene. It has an estimated prevalence of 1 in 5000 males and 1 in 8000 females (Tassone et al., 2012). This expansion typically results in methylation of the gene, leading to gene silencing and little or no production of the *FMR1* protein (FMRP), resulting in the clinical features of FXS, including intellectual disability (Bagni et al., 2012; Saldarriaga et al., 2014). The *FMR1* gene premutation (PM) with 55–200 CGG repeats commonly occurs in approximately 1 in 400 males and 1 in 200 females (Cabal-Herrera et al., 2020; Tassone et al., 2023). Aging individuals with the PM may develop Fragile X-associated Tremor/Ataxia Syndrome (FXTAS), which may present with intention tremor, cerebellar ataxia, cognitive decline, and brain changes detected via magnetic resonance imaging (MRI) (Hagerman and Hagerman, 2016; Famula et al., 2022). These changes include white matter hyperintensities (WMHs) in the middle cerebellar peduncles (MCP sign), splenium, and/or periventricular areas (Hagerman and Hagerman, 2016; Hall et al., 2017). Current studies on FXTAS have focused on the impact of the premutation on cognitive abilities, particularly executive functioning (EF), and memory issues as an early manifestation of FXTAS (Cabal-Herrera et al., 2020; Famula et al., 2022; Hessl et al., 2024).

FXTAS patients experience mRNA toxicity, leading to issues such as protein sequestration, DNA damage, and repeat-associated non-AUG (RAN) translation which results in cell death, particularly of astrocytes and neurons (Glineburg et al., 2018; Dufour et al., 2024). The diagnostic criteria for FXTAS are based on a combination of clinical features (tremor and ataxia) in addition to characteristic radiological features, including WMHs in the MCP and in the splenium of the corpus callosum (Hall et al., 2014). Both the clinical presentation and these associated radiological findings are more commonly observed and tend to be more severe in male carriers (Hagerman and Hagerman, 2016).

By their 70s, about 48% of male PM carriers develop FXTAS. In those aged 80 years and above, this number increases to 75% (Jacquemont, 2004; Coffey et al., 2008; Rodriguez-Revenga et al., 2009). However, clinical presentation can vary significantly in terms of severity, age of onset, and overall course (Jacquemont, 2004; Hagerman and Hagerman, 2016; Famula et al., 2018; Zhao et al., 2020). Since the gene responsible for FXTAS is X-linked, it affects males and females differently. Female carriers tend to have lower penetrance and milder clinical symptoms compared to males because of the protective effects of their second X chromosome (Rodriguez-Revenga et al., 2009; Schneider et al., 2020). Symptom severity in female PM carriers may be related to X-inactivation patterns that favor higher expression of the normal allele for the protective effect (Berry-Kravis et al., 2005). Recent studies have explored biomarkers that aid in identifying carriers at higher risk of developing FXTAS (Renaud et al., 2015; Shelton et al., 2018; Zafarullah et al., 2020a, 2020b). Despite these efforts, clinical measures to assess individual risk or early disease detection are still limited. Clinical heterogeneity persists after disease onset, where some patients experience a sudden increase in symptom severity, especially with concomitant excessive alcohol intake or opioid use (Muzar et al., 2014), while in others, the disease progresses more gradually (Leehey, 2009; Robertson et al., 2016).

Researchers have extensively studied the neuropsychological profiles of PM carriers through cross-sectional approaches and have found that even among carriers with FXTAS symptoms, subtle cognitive effects may be present before motor signs develop; these effects are linked to white matter changes in the brain (Filley et al., 2015; Loesch et al., 2024). Recent well-powered brain volumetric studies suggest that the disease process starts earlier than when formal signs of FXTAS are detected (Cabal-Herrera et al., 2020; Wang et al., 2020). Building upon the understanding of FXTAS variability and its early cognitive effects, our study aims to assess EF using the nine-item performance-based version of the Behavioral Dyscontrol Scale (BDS-2), to complement instrument based measures of disease severity (Grigsby and Kaye, 2020).

The Behavioral Dyscontrol Scale (BDS) measures EF through various domains of behavior and attention (Grigsby and Kaye, 1996). The first version of BDS was created to test the executive functioning of geriatric populations, which showed a ceiling effect when the patient demographic included younger participants (Shura et al., 2015). This limitation was addressed by BDS-2, which reduced negative skewness in samples ranging from ages 18 to 71 (Leahy et al., 2003). While some studies utilized sensor-based systems such as Kinesia to track patients’ tremors (Park et al., 2019), this study used BDS-2 to evaluate executive dysfunction and its relationship with neuroimaging markers for disease severity in FXTAS (Grigsby and Kaye, 2020).

Similar to BDS-2, many other cognitive tests have been developed to measure EF. Executive functioning refers to a set of cognitive processes that enable the management and regulation of behavior effectively. Research has found that males with the PM experience a greater decline throughout their lifetime in their EF, compared to females with the PM (Cornish et al., 2009). This sex-specific divergence is hypothesized to be driven by the X-chromosome inactivation in female PM carriers as they possess a second X chromosome expressing the normal allele, which is thought to provide a protective buffer against the severe executive deficits observed in males. This is supported by multiple studies demonstrating that females have milder deficits from FXTAS compared to males (Jacquemont, 2004; Schneider et al., 2020). Furthermore, because executive dysfunction predates the onset of classical motor symptoms of FXTAS, consequently, studying EF may facilitate earlier detection and intervention (Hessl et al., 2025). Here, we report the correlations between BDS-2, executive functioning, and the severity of the WMH on MRI.

## 2 Methods and materials

### 2.1 Study design and participants

This is a cross-sectional study of 56 p.m. carriers diagnosed with FXTAS as part of the NIH-funded longitudinal genotype–phenotype study of Fragile X families conducted at the UC Davis MIND Institute (NICHD HD036071). For this cross-sectional analysis, we included males and females, aged 44–86 years at the time of the evaluation, meeting the following criteria:

1) Confirmed *FMR1* PM carriers without clinical diagnosis of FXTAS (stage 0). Participants with missing CGG repeat values were recruited into the study as PM carriers based on prior molecular testing performed by outside laboratories.
2) Participants with clinical diagnosis of FXTAS (Stage 1–6, though the final sample contained no individuals at Stage 6).
3) Participants who completed all nine items of the BDS-2 assessment to provide a complete BDS-2 total score.
4) Participants underwent complete structural brain MRI with semiquantitative scores (0–3) for cerebral atrophy, cerebellar atrophy, and WMH burden, plus binary scores for genu WMH and corpus callosum thickness (0–1) across all tracked regions in FXTAS to generate a total MRI score.

All participants provided informed consent according to the Declaration of Helsinki, and the project was approved by the University of California, Davis Institutional Review Board. Participants underwent neuropsychological testing and MRI imaging during the same study visit.

### 2.2 Neuropsychological testing

**Figure 1:**
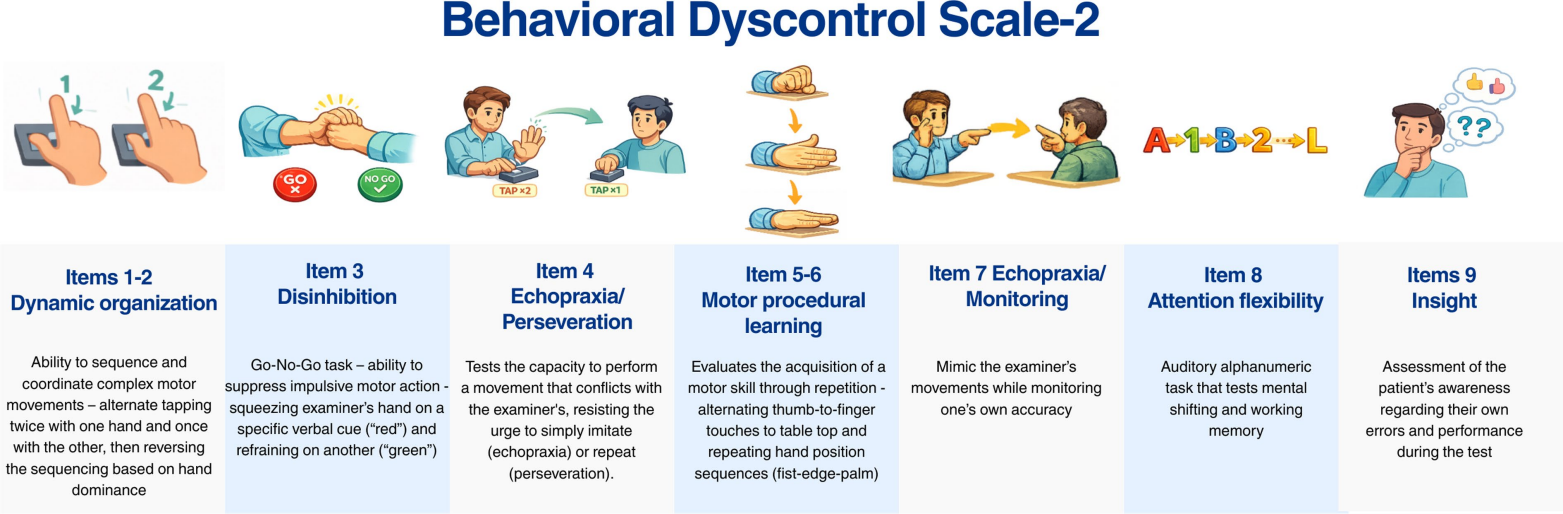
Components of the Behavioral Dyscontrol Scale-2 (BDS-2). The scale consists of nine performance-based items assessing executive self-regulation, with each item scored from 0 to 3 and a total score ranging from 0 to 27.

BDS-2 is a brief 9-item performance-based assessment of frontal-subcortical EF that measures intentional self-regulation, motor control, behavioral inhibition, sequencing, and working memory (Grigsby and Kaye, 1996). Each item is scored 0–3 with a total score of 0–27, with lower scores indicating higher executive dysfunction. The 9-item subtests are summarized in Figure 1.

Participants also underwent cognitive testing for the Mini-Mental State Examination (MMSE) and the executive subtests of the Cambridge Neuropsychological Test Automated Battery (CANTAB), Spatial Working Memory (SWM), One Touch Stockings of Cambridge (OTS), and Stop Signal Task (SST) (Fray et al., 1996). The SWM subtest measures the ability to retain spatial information and manipulate it in working memory. The goal for the participant is to find tokens hidden inside colored boxes on the screen to fill a sidebar. Once a token is found, that box will never contain a token again during that round, and the participant must remember which boxes have already been emptied. The OTS subtest measures spatial planning and executive decision-making. In this task, two displays of three colored balls held in “stockings” are displayed with one display being the “goal” pattern and another being the “starting” pattern. The participant is required to determine the minimum number of moves required to make the “starting” pattern match the “goal” pattern. The participant selects a number at the bottom of the screen representing the calculated solution of minimum number of moves. The initial iteration is solved with one move, and the number of moves increases in subsequent problems. The SST measures response inhibition. It assesses a construct similar to that measured by Go/No-Go tasks with the participant responding quickly to the “go” stimulus in terms of direction of arrows (left-hand button for left pointing arrow and right-hand button for right pointing arrow) and inhibiting their response once the auditory “stop signal” appears. The estimated time required to inhibit the response is calculated as the Stop Signal Reaction Time (SSRT).

### 2.3 MRI imaging

Participants also underwent MRI imaging during the same visit to generate a quantitative score of 0–3. All MRI scoring protocols were performed by a single, trained rater who was blinded to clinical data. MRI ratings included cerebral and cerebellar atrophy, as well as WMH burden in the cerebral, cerebellar, middle cerebellar peduncle, pons, sub-insular, periventricular, and splenial regions. Scores were defined as 0 = none, 1 = mild, 2 = moderate and 3 = severe. Genu WMH and corpus callosum thickness were scored as binary variables (0 = absent/normal, 1 = present). This resulted in a continuous total score of 0–29 with higher scores indicating greater neurodegenerative involvement.

### 2.4 Clinical progression of FXTAS

**Figure 2:**
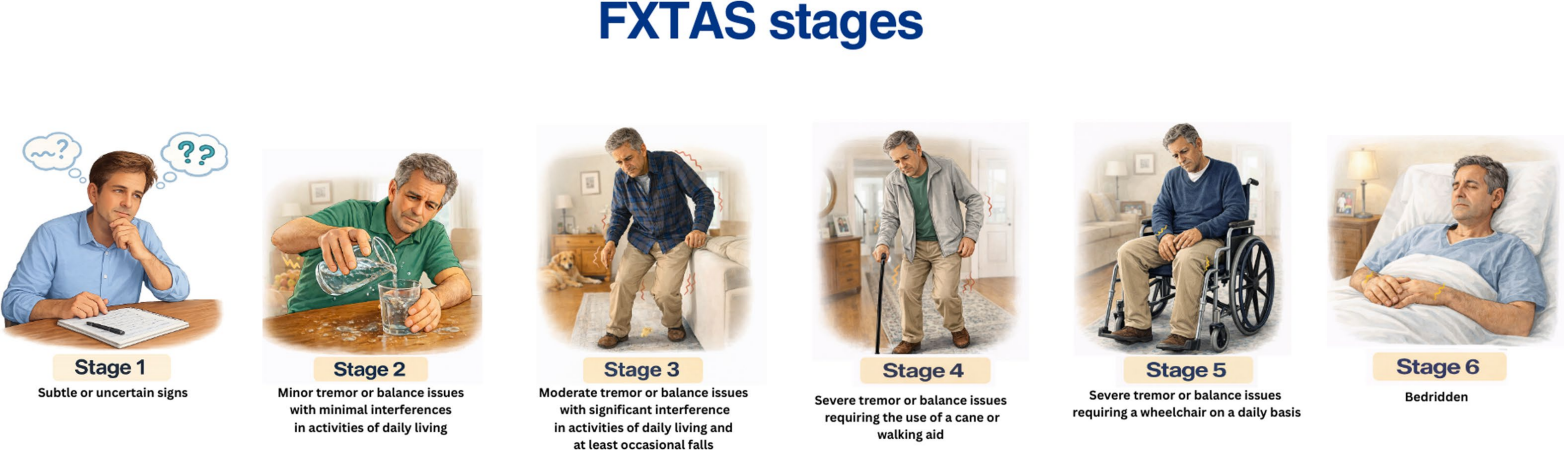
Clinical staging framework for Fragile X-associated Tremor/Ataxia Syndrome (FXTAS), illustrating progression from premutation carriers without FXTAS (Stage 0) through severe disease (Stage 6).

FXTAS disease severity was classified on the basis of established clinical staging criteria (1–6) (Bacalman et al., 2006). FXTAS stages are illustrated in Figure 2.

### 2.5 CGG repeat sizing

Genomic DNA (gDNA) was isolated from 3 mL of whole blood using standard procedures. CGG repeat allele size and methylation status were determined by using Southern blot and PCR analysis. Details of the protocols are as previously reported (Tassone et al., 2008; Filipovic-Sadic et al., 2010).

### 2.6 Statistical analysis

Analyses were performed using R (version 4.5.2; R Foundation for Statistical Computing, Vienna, Austria). Descriptive statistics for participant characteristics were summarized using means (SD), medians (ranges) and frequencies (%). Normality and homoscedasticity were assessed using the Shapiro–Wilk test and Levene’s test for the assumptions of a parametric test. When the assumptions of a parametric test were not satisfied, a non-parametric equivalent test was used as appropriate. Basic characteristics between male and female participants were compared using Wilcoxon rank-sum tests for continuous variables and Fisher’s exact test for categorical variables. Differences in BDS-2 scores across FXTAS stages were examined using a Kruskal-Wallis test, followed by Dunn’s *post hoc* tests to determine which specific FXTAS stages differed in BDS-2 performance. Correlation analyses were performed using Pearson’s or Spearman’s rank correlations as appropriate. The primary association between BDS-2 and MRI total scores was assessed using linear regression models to account for covariates. Linear regression assumptions (linearity, normality of residuals, and homoscedasticity) were examined through analysis of residuals using scatterplots, LOESS curves, Q-Q plots, and scale-location plots. Sensitivity analyses of the influence of extreme values of CGG repeats were conducted, and one observation (CGG = 185) was identified and excluded from linear regression analysis. Age, sex, and CGG repeat length were included as covariates in regression models for covariate-adjusted analyses. To examine the correlations between neuropsychological tools (BDS-2, MMSE and CANTAB subtests) with MRI total scores, correlation analyses were restricted to a subset of participants with complete data across all measures (*n* = 30). Statistical significance was determined at a two-sided *p* ≤ 0.05.

## 3 Results

Descriptive statistics of study participants are summarized in Table 1. The study sample included 57 participants, of whom 35 (61%) were male and 22 (39%) were female. The mean age at visit was 68 ± 9 years. Males had significantly worse MRI total scores (*p* < 0.001) and lower total BDS-2 scores (*p* = 0.001) as expected because females with FXTAS are less severely involved compared to males in both cognition (Seritan et al., 2013) and MRI findings (Schneider et al., 2020). There were no significant sex differences in CGG repeat length, MMSE, CANTAB subtests (SWM and OTS), and FXTAS stage.

**Table 1:** Demographics of patient characteristics of study sample.

Characteristic	Overall *N* = 57	Male *N* = 35	Female *N* = 22	Male vs. Female *p*-value
Age at visit				0.4^1^
Mean (SD)	68 (9)	69 (7)	67 (11)	
Median [min, max]	67 [44, 86]	68 [55, 86]	66 [44, 85]	
Primary race, *n* (%)				0.64^2^
Asian	2 (4.1%)	1 (3.2%)	1 (5.6%)	
Other^3^	1 (2.0%)	1 (3.2%)	0 (0%)	
Unknown/not reported	1 (2.0%)	0 (0%)	1 (5.6%)	
White	45 (92%)	29 (94%)	16 (89%)	
Missing	8	4	4	
BDS2 Total Score				0.001^1^
Mean (SD)	20.0 (4.4)	18.6 (4.8)	22.3 (2.4)	
Median [min, max]	20.0 [4.0, 27.0]	20.0 [4.0, 27.0]	23.0 [17.0, 26.0]	
MRI Total Score				<0.001^1^
Mean (SD)	11 (7)	14 (6)	7 (4)	
Median [min, max]	11 [1, 25]	14 [2, 25]	7 [1, 15]	
CGG				0.3^1^
Mean (SD)	89 (20)	92 (22)	85 (18)	
Median [min, max]	88 [52, 185]	90 [61, 185]	81 [52, 119]	
Missing	1	1		
MMSE				0.056^1^
Mean (SD)	27.65 (3.08)	26.97 (3.70)	28.64 (1.40)	
Median [min, max]	29.00 [13.00, 30.00]	28.00 [13.00, 30.00]	29.00 [26.00, 30.00]	
Missing	3	3	0	
SWM				0.019^1^
Mean (SD)	21 (12)	24 (13)	15 (8)	
Median [min, max]	22 [0, 71]	23 [0, 71]	17 [0, 27]	
Missing	20	10	10	
SST				<0.001^1^
Mean (SD)	649 (93)	684 (82)	579 (73)	
Median [min, max]	630 [464, 845]	703 [538, 845]	580 [464, 696]	
Missing	24	13	11	
OTS				0.2^1^
Mean (SD)	2.15 (0.85)	2.25 (0.90)	1.93 (0.75)	
Median [min, max]	1.77 [1.13, 4.33]	1.87 [1.33, 4.33]	1.60 [1.13, 3.20]	
Missing	23	12	11	
FXTAS Stage (0–5), *n* (%)				0.14^2^
0	2 (3.6%)	0 (0%)	2 (9.5%)	
1	2 (3.6%)	0 (0%)	2 (9.5%)	
2	16 (29%)	9 (26%)	7 (33%)	
3	19 (34%)	14 (40%)	5 (24%)	
4	14 (25%)	10 (29%)	4 (19%)	
5	3 (5.4%)	2 (5.7%)	1 (4.8%)	
Missing	1	0	1	

^1^Wilcoxon rank sum exact test.

^2^Fisher’s exact test.

^3^Australian aboriginal.

### 3.1 Correlation between BDS-2 scores and FXTAS stages

**Figure 3:**
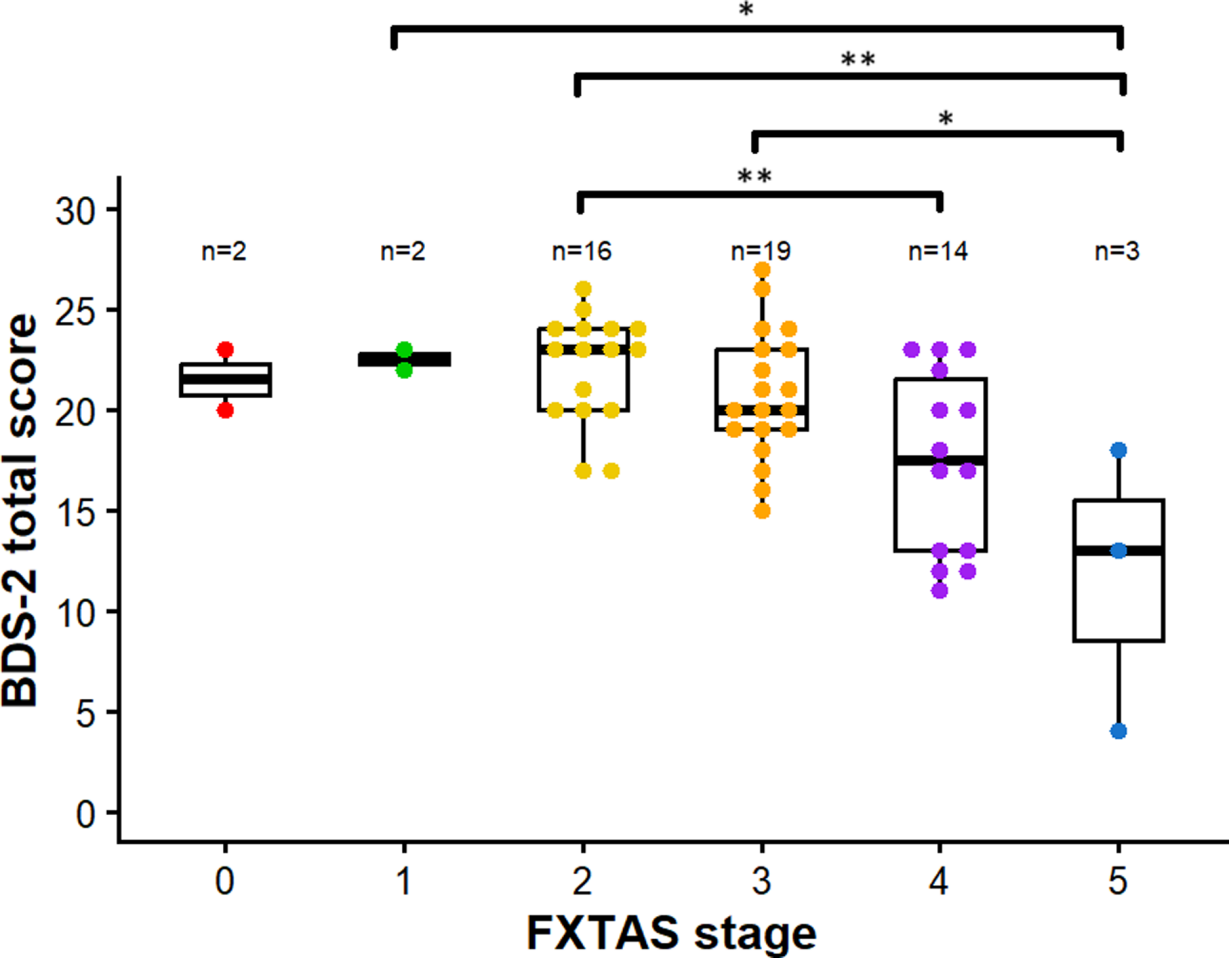
Distribution of BDS-2 total scores across FXTAS clinical stages. Stage 0 represents premutation carriers without a clinical diagnosis of FXTAS. Dunn’s *post hoc* tests were conducted for pairwise group comparisons: *p < 0.05 and **p < 0.01.

Significant differences in BDS-2 total scores across FXTAS stages (Kruskal-Wallis, *p* = 0.0087, n = 56) were observed. Dunn’s *post hoc* tests showed significant pairwise differences in BDS-2 scores between Stages 1 and 5 (*p* = 0.0436); 2 and 4 (*p* = 0.0020); 2 and 5 (*p* = 0.0034); and 3 and 5 (*p* = 0.0284) (Figure 3), showing lower BDS-2 total scores in more advanced FXTAS stages.

### 3.2 Relationship between BDS-2 and MRI scores

**Figure 4:**
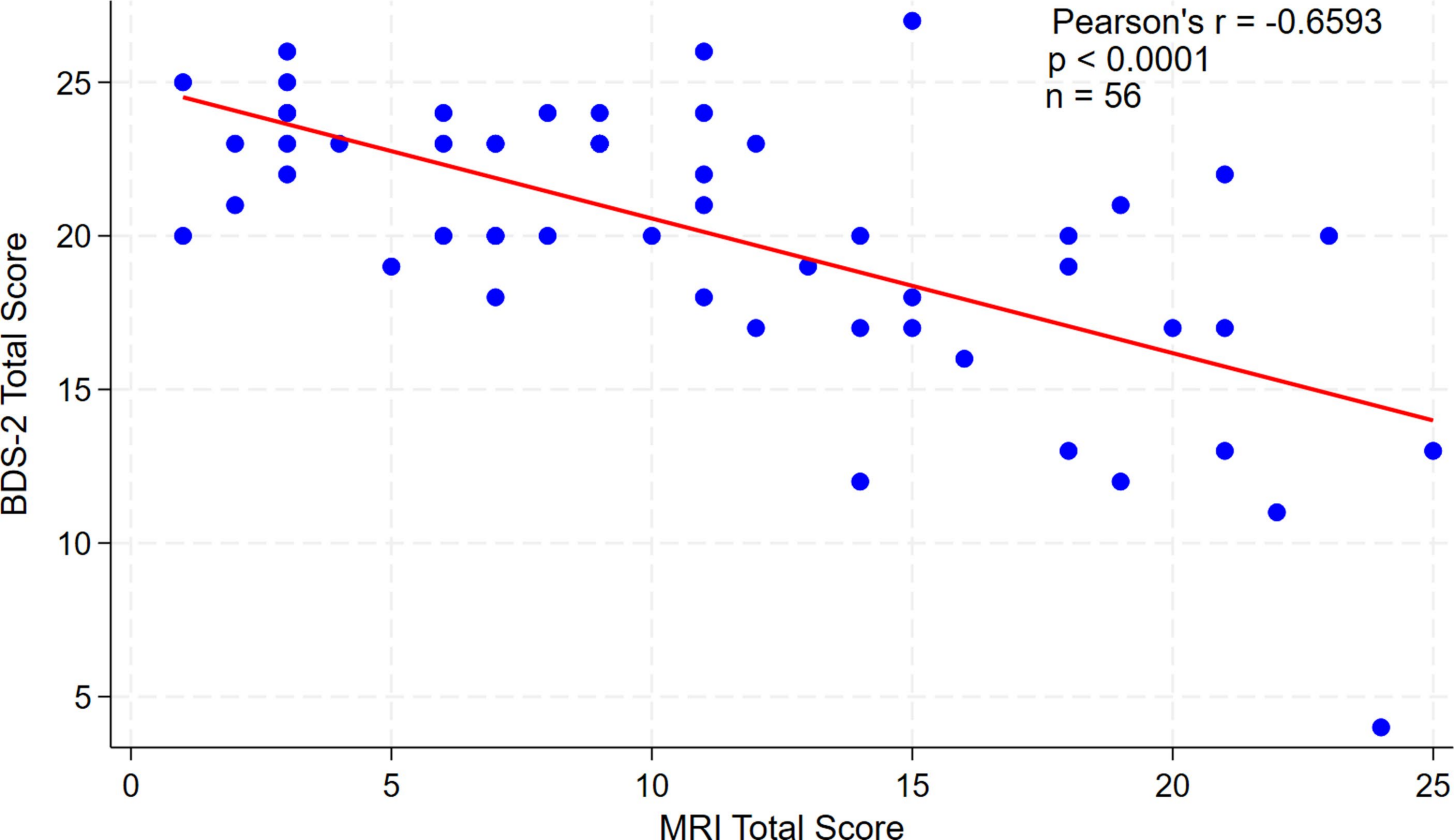
Correlation between MRI total scores and BDS-2 total scores. Each dot represents an individual participant. The solid line represents the fitted regression line.

MRI score was negatively correlated with BDS-2 total score for all participants (Pearson’s *r* = −0.66, *p =* < 0.0001) (Figure 4). Linear regression analysis was conducted to evaluate the association between MRI total score and BDS-2 total score while adjusting for CGG repeat length, sex, and age at visit as covariates in the linear model. We excluded an extreme outlier of CGG = 185 from this analysis. MRI total score remained significantly associated with BDS-2 total score (*β* = −0.36, *p* = 0.002) when adjusted for CGG repeat length, sex, and age.

### 3.3 Correlation of neurocognitive measures with MRI

We conducted this correlation analysis using the complete-case sample who completed all assessments (*n* = 30). The executive subtests of the CANTAB demonstrated positive correlations with MRI total score at similar degrees such as the SST (Spearman’s *ρ* = 0.57, *p* = 0.0014), OTS (*ρ* = 0.61, *p* = 0.0005), and SWM (*ρ* = 0.46, *p* = 0.011). The global MMSE score showed a negative correlation with the MRI total score (*ρ* = −0.40, *p* = 0.03).

### 3.4 Criterion validity of BDS-2

**Figure 5:**
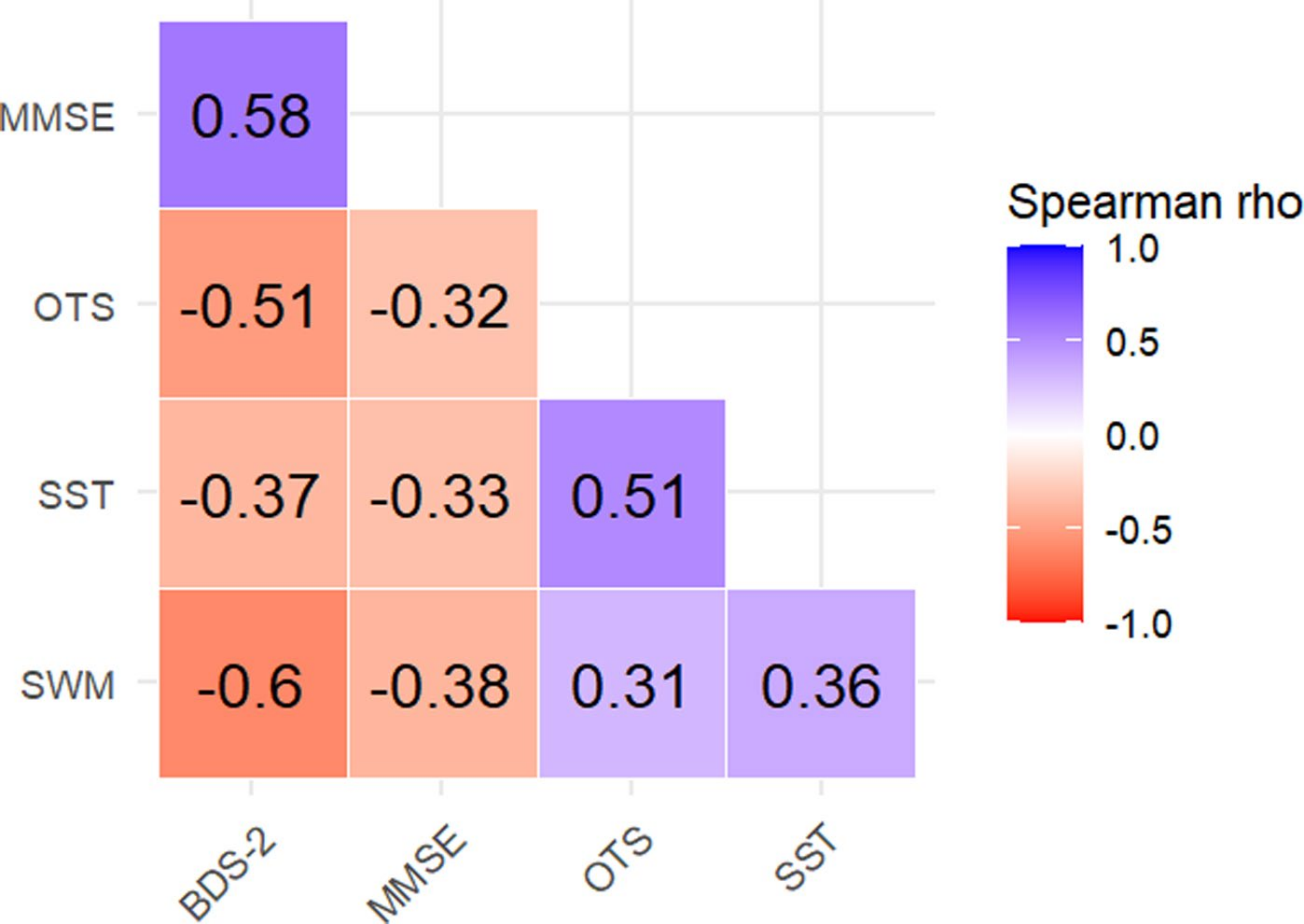
Correlation heatmap matrix showing Spearman correlation coefficients among BDS-2, MMSE, and CANTAB executive function measures, including the Stop Signal Task (SST), Spatial Working Memory (SWM), and One Touch Stockings of Cambridge (OTS) (*n* = 30).

Criterion validity was assessed by examining correlations between BDS-2 and established cognitive measures—MMSE and CANTAB subtests (SST, OTS, and SWM) (Figure 5). Within the complete-case sample (*n* = 30), a strong positive correlation between BDS-2 and MMSE was observed (*ρ* = 0.58, *p* = 0.0009), suggesting that higher executive functioning is associated with better global cognitive performance. This is expected clinically and supports criterion validity. In addition, BDS-2 as an executive function measure was negatively correlated with CANTAB-2 working memory performance (SWM, *ρ* = −0.60, *p* = 0.0006), worse planning (OTS, *ρ* = −0.51, *p* = 0.0045), and reaction time (SST, *ρ* = −0.37, *p* = 0.044).

To further evaluate clinical relevance, associations with FXTAS clinical stage were examined. BDS-2 significantly correlated with FXTAS stage (*ρ* = −0.50, *p* = 0.0001). Comparatively, significant correlations with FXTAS stage were also observed for the MMSE (*ρ* = −0.54, *p <* 0.0001), CANTAB SST (*ρ* = +0.51, *p* = 0.003), and CANTAB OTS (*ρ* = +0.60, *p* < 0.0001), while CANTAB SWM was not significant (*ρ* = +0.25, *p* = 0.15). Higher scores on CANTAB SWM, SST, and OTS indicate poorer working memory, response inhibition, and planning performance respectively; thus, negative correlations indicate that superior executive self-regulation on the BDS-2 is consistently linked to better objective cognitive task execution.

## 4 Discussion

The clinical significance of the *FMR1* PM has evolved substantially over the past three decades. Once considered a largely asymptomatic carrier state, the identification of Fragile X-associated primary ovarian insufficiency (FXPOI) and FXTAS established that PM carriers are at increased risk for a range of neurodevelopmental, neuropsychiatric, reproductive, and neurodegenerative manifestations (Cronister et al., 1991; Sullivan et al., 2005; Hagerman et al., 2001). Tassone et al. first reported elevated *FMR1* mRNA levels in those with the PM (Tassone et al., 2000) which is believed to contribute to RNA toxicity, neuronal and glial dysfunction, and the development of WMHs and cerebral atrophy (Tassone et al., 2023). The finding of developmental deficits in some PM carriers as early as infancy or early childhood (Farzin et al., 2006; Gallego et al., 2014; Aishworiya et al., 2022), and a cross-sectional study of CNS changes throughout life in carriers versus controls (Wang et al., 2017) demonstrates that the RNA toxicity of the PM can impact the brain well before the onset of FXTAS.

Executive dysfunction is among the earliest and most consistently reported cognitive changes observed in PM carriers and often precedes the emergence of the motor features required for clinical diagnosis of FXTAS (Famula et al., 2022; Hessl et al., 2024). In recent treatment studies, including an intravenous allopregnanolone trial in FXTAS (Wang et al., 2017; Napoli et al., 2019) and an oral sulforaphane study (Santos et al., 2023), aspects of EF have been shown to improve. Therefore, EF measures may represent particularly relevant neuropsychological outcome measures in clinical trials.

Traditionally, diagnosing and monitoring FXTAS have relied on clinical observations and neurological examinations. The primary finding of this study is that lower BDS-2 performance was associated with greater MRI-defined neurodegenerative burden in individuals with FXTAS. Given that the BDS-2 assesses self-regulation, sequencing, inhibitory control, motor regulation, and other executive processes that rely on frontal-subcortical circuitry, the observed association is biologically plausible within the context of the widespread white matter and cerebellar pathology characteristic of FXTAS. Because the BDS-2 is a simple and rapid tool that correlates with MRI-defined disease severity, the BDS-2 is highly practical for clinical follow-up and the assessment of cognitive decline associated with FXTAS.

The BDS-2 was originally developed to assess behavioral dyscontrol arising from dysfunction within frontal-subcortical systems and has demonstrated sensitivity to deficits in self-regulation, response inhibition, sequencing, working memory, and goal-directed behavior (Shura et al., 2015). These domains overlap closely with the cognitive processes known to be vulnerable in FXTAS, supporting the conceptual relevance of the instrument for this population.

BDS-2 may capture these specific vulnerabilities more effectively than broader screening tools such as the MMSE due to its focal sensitivity to frontal-subcortical pathways (Bacalman et al., 2006). While the MMSE serves as a global cognitive screening tool heavily weighted toward language and orientation, it frequently exhibits ceiling effects in early-stage neurodegenerative diseases and lacks the granularity required to evaluate specific motor-regulation, sequencing, and inhibitory controls (Shura et al., 2015). Compared with broad cognitive screening measures such as the MMSE, the BDS-2 specifically targets executive and behavioral self-regulatory processes that are thought to be particularly vulnerable in FXTAS. Consistent with this conceptual distinction, both BDS-2 and MMSE scores were associated with MRI severity; however, the BDS-2 may provide complementary information regarding executive dysfunction and functional self-regulation that is not directly assessed by global cognitive screening instruments. Because executive dysfunction may represent one of the earliest cognitive manifestations of FXTAS, a bedside tool assessing self-regulation provides clinical utility for identifying early functional impairment (Famula et al., 2022; Hessl et al., 2024). However, because our present study design is cross-sectional, these associations are strictly limited to characterizing disease severity rather than demonstrating direct longitudinal progression within individuals, which warrants future longitudinal studies.

While BDS-2 has demonstrated utility in assessing EF, we can further refine its suitability for FXTAS patients. Future studies should examine the relative contribution of individual BDS-2 items to overall disease severity. Item-level analyses may help identify whether specific domains, such as inhibitory control, motor sequencing, working memory, or behavioral regulation, are particularly sensitive to early FXTAS-related changes. Such information could support the development of more targeted clinical outcome measures and improve understanding of the cognitive phenotype associated with FXTAS.

Beyond BDS-2, we incorporated other neuropsychological tests, measured by the CANTAB subtests, to examine specific components of cognition including visual and spatial memory (Al Backer et al., 2019). We additionally compared BDS-2 performance with established computerized executive function measures from the CANTAB battery. Correlations between MRI severity and CANTAB measures were of similar magnitude to those observed for the BDS-2, supporting convergent validity across multiple approaches to executive function assessment. Importantly, whereas CANTAB testing requires specialized computerized administration, the BDS-2 can be completed rapidly at the bedside, suggesting potential practical advantages for routine clinical monitoring and research settings. These findings complement recent longitudinal neuroimaging work by Hessl et al. (2025), which demonstrated progressive brain volume loss and increasing white matter pathology over approximately 1.3 years in individuals with FXTAS. Together, these studies support the concept that structural neurodegeneration progresses measurably over time and that executive dysfunction may serve as a clinically meaningful correlate of these underlying brain changes. Our study aimed to expand on these findings by exploring whether BDS-2 items, CANTAB, and MMSE are associated with MRI severity and FXTAS stages.

### 4.1 Limitations

Several limitations of this study should be noted. First, the cross-sectional design prevents the assessment of longitudinal changes in EF and neurodegeneration within individuals. Further longitudinal studies are warranted to evaluate the utility of BDS-2 in projecting disease progression over time. Second, data for specific cognitive subtests, such as the CANTAB, were not available for all 57 participants. Missing CANTAB data were primarily attributed to strict logistical time constraints during the study visit or the premature termination of the test due to participant frustration or severe physical tremors. Therefore, correlation analyses between CANTAB subtests and MRI scores were limited to a small subset of the participants with all complete measures in this study. Third, our cohort was predominantly White (92%), which may limit the generalizability of these findings to more diverse populations. In addition, a single rater scored MRI scores for all participants in this study and thus it is necessary to evaluate intra and inter-rater reliability across multiple raters, which is desired in future studies to confirm scoring protocol reproducibility. Because this study was exploratory in nature, we did not control multiple comparisons for multiple measures on the same set, inflating the risk of a Type I error and thus cautionary interpretation is needed. Our results demonstrated significant sex differences in BDS-2 and MRI scores, with female participants exhibiting higher BDS-2 scores and lower MRI severity scores compared to males. These findings are consistent with the generally lower FXTAS burden reported in females compared to males across both structural and behavioral domains, consistent with the BDS-2 being sensitive to known sex-specific variations in FXTAS burden. However, this study did not evaluate sex-specific psychometric properties due to the small sample size, which warrants future investigation in larger stratified cohorts.

## 5 Conclusion

Our study supports the use of the BDS-2 as a clinically relevant measure associated with disease severity in individuals with FXTAS. The BDS-2 demonstrated strong associations with MRI markers, FXTAS stage, and established cognitive measures. When used to complement traditional MRI assessments, BDS-2 offers a comprehensive evaluation of cognitive function across multiple domains. Collectively, these findings support further investigation of the BDS-2 as a practical and accessible measure of executive dysfunction in FXTAS and as a potential candidate outcome measure for future longitudinal and interventional studies.

### 5.1 Future directions

While this study demonstrates a strong association between BDS-2 performance and objective MRI severity markers, a rigorous assessment of the clinical utility of the BDS-2 in FXTAS has not yet been established. To transition this tool successfully into clinical trials, future longitudinal investigations are strongly warranted. Specifically, prospective trials must establish test–retest reliability, responsiveness to clinical change, and the minimal clinically important difference (MCID) across various FXTAS stages. Defining these parameters is a critical next step in determining the suitability of the BDS-2 as a therapeutic endpoint.

## Data Availability

The data analyzed in this study is subject to the following licenses/restrictions: the datasets analyzed for this study are not publicly available due to institutional policy and patient confidentiality requirements. Access to the raw data is restricted to protect the privacy of the participants in accordance with HIPAA regulations and the UC Davis Institutional Review Board. Data may be made available to qualified researchers upon reasonable request and with appropriate ethical approval. Requests to access these datasets should be directed to RH, rjhagerman@ucdavis.edu.
